# Immigrants Serving in Local Government: A Systematic Review and Meta-Analysis of Factors Affecting Candidacy and Election

**DOI:** 10.1177/10780874211038500

**Published:** 2021-08-18

**Authors:** Shervin Ghaem-Maghami, Vincent Z. Kuuire

**Affiliations:** 1 Department of Geography, Geomatics, and Environment, 71637 University of Toronto Mississauga, Mississauga, Canada; 2 Social and Behavioural Health Sciences Division, Dalla Lana School of Public Health, University of Toronto, Toronto, Canada

**Keywords:** political representation, immigrants, candidates, elections, local government

## Abstract

Descriptive representation, the extent to which politicians reflect the descriptive characteristics (e.g., ethnicity or gender) of their constituents, has been studied at various scales since it was first introduced in Hanna Pitkin's seminal work several decades ago. In recent years, scholars have also begun to investigate immigrant representation in politics, including at the local, state, and national levels of government. This study evaluates the current research on the factors affecting the election of immigrant candidates to municipal government. In addressing the lack of data-driven reviews in this type of research, the paper employs a scoping review methodological framework. Fifty-six distinct factors are identified as important for immigrants’ electoral fortunes. The factors are classified under: Macro-level electoral structures and situational elements, meso-level immigrant group dynamics, and micro-level individual candidate characteristics. The most salient factors are elaborated on, together with a discussion on policy implications and future potential areas of inquiry.

## Introduction

There is a general consensus that, despite making up an ever-increasing proportion of the populations of most major cities in the world (Nicholls and Uitermark 2016), immigrants and refugees are largely under-represented in elected parliaments and other types of public decision-making bodies (Bird 2005). The mediascape is replete with accounts of unwieldy waves of newcomers arriving at the borders of relatively prosperous nations that are unwilling to welcome them. In the highly politicized climate of the present day, these stories often underplay or ignore entirely the considerable degree of agency that migrants must exercise in moving along precarious routes to reach and traverse the national borders of their countries of destination. Further, subsequent to their arrival, they must draw upon an additional reserve of resourcefulness to navigate complex legal and physical structures in the hope of improving their living conditions and those of their families (Sigona 2012). This study asserts that exercising agency, a strength that is inherently at immigrants’ disposal, does not stop following successful relocation and settlement; rather, it remains central to continuing their trajectory toward full integration into their host societies—including in achieving political representation.

The recent political experiences of some immigrants in North America shed light on the intricate connections between migrant agency and the countless structural factors that are exerting influence on and shaping the political landscape. For example, when Wilmot Collins escaped the Liberian Civil War in the early-1990s and reached Montana in the United States as a refugee, it was likely unimaginable in his eyes to envision himself as the first Black person to be elected the mayor of any city in the history of the state within 25 years of his arrival. In 2017, Collins defeated the four-term incumbent mayor of the city of Helena with little support to draw upon from his own ethno-racial community, a strategy that is commonly used by immigrant candidates to establish a solid electoral base; the combined Black, African American, and mixed-race population of the city was under 4% at the time (U.S. Census Bureau 2019). Within 2 years of his mayoral victory, Collins had already launched a campaign for the U.S. Senate, describing his method of outreach as aiming to “meet as many people as possible and make connections on common issues such as trade and student loan debt” (Siegler 2019).

Only 3 years earlier across the border in Canada, Olivia Chow was the only woman, ethnic minority, and immigrant candidate among the three major contenders in the 2014 mayoral election in Toronto, Ontario (Bird et al. 2016). Chow's prospects were hopeful, given that she had served as a popular member of parliament representing the social democratic party in a downtown Toronto ward for 8 years prior to the election, and she became the instant front-runner following her announcement to join the race to become the city's mayor (Maiolino 2018). Yet, despite being among the most multicultural cities in the world, Toronto has a historically poor record of electing immigrants and minorities to public office (Siemiatycki 2011). While evidence was later found of a strong inter-ethnic affinity effect among minorities in support of Chow (Bird et al. 2016), she would nevertheless end up with only 23% of the popular vote and finish last out of the three principal candidates.

Although these are just illustrations of two isolated examples, there are lessons that can be gleaned from these experiences, and this can be extended to all studies of elections featuring immigrant candidates vying for positions in local government. Any effort to try to appreciate the complex circumstances under which Collins, Chow, and the many other immigrant candidates who have sought municipal public office naturally points to the need for a meta-analysis to determine the broad range of factors that could possibly influence their electoral outcomes and, in a more general sense, come to bear upon effective political representation.

A fundamental element of all vibrant democracies is that representation in the political realm reflects the composition of a given society's membership. Yet, in light of rapid changes in the ethnocultural composition of host societies and ever-evolving ideas around who is deserving of the privilege to represent a population, clarity regarding *how* representation works and *how it should* work remain elusive. The touchstone for most research pursuing these lines of inquiry is Pitkin’s (1967) *The Concept of Representation*. Her designation of “descriptive representation,” defined as the extent to which politicians reflect the descriptive characteristics (e.g., ethnicity or gender) of the communities they serve, remains relevant more than 50 years later in contemporary research on voter behavior, pre-election political strategy, and the likelihood of a candidate's success. Available evidence demonstrates that from the perspective of immigrant communities, co-ethnic politicians benefit from a superior understanding of their unique concerns because they have similar backgrounds and experiences. Beyond creating an opening for the community to access the political system, having one's “own” politicians represented in government is a symbolic indication of a given community's importance, both within and outside of the immigrant population (Bloemraad 2006).

Achieving descriptive representation appears to depend in part on immigrant integration into the host society, which is facilitated by that society's acceptance of changing demographics and evolving local structural factors. Local factors in particular, such as the presence of left-leaning governments and a strong network of community-based organizations, have a direct impact on how integration policies emerge and how they are operationalized (de Graauw and Vermeulen 2016). Among these factors and their associated practices are those that create opportunities to promote newcomers’ increased incorporation into political life, the lack of which can dampen enthusiasm even for those who may bring an abundance of experience with them from participating in civic activities and associations in their countries of origin (Voicu and Rusu 2012). In this light, one could anticipate that the most outstanding factors contributing to the election of immigrant-background candidates to local government might be the degree to which candidates have achieved socio-economic integration into the local context of their host societies and the concentration of immigrant communities within electoral jurisdictions as it relates to voter turnout.

An invaluable overview of some of the noteworthy factors in immigrant political representation is provided by Bloemraad and Schönwälder (2013), who present both the conceptual challenges and theoretical approaches to evaluating migrant and ethnic minority representation. Among the conceptual challenges are inconsistencies in terms of the populations being studied and compared—e.g., first- or second-generation “immigrants,” lumping together all national or ethno-linguistic “minorities,” a given religious community versus ethno-racial groups that also belong to the same religious organization, or failing to acknowledge the nuances affecting each group's unique trajectory of integration. Bloemraad and Schönwälder (2013) propose a “new institutionalism perspective” that emphasizes interactions between individual and collective actors and institutional and cultural contexts. Further, they indicate that there is an absence of comparative studies of immigrant political incorporation, often owing to inadequate secondary data collection on ethnicity or migration status, as well as a lack of systematic overviews of immigrant and ethnic minority representation in local, regional, and national political bodies. Garbaye and Mollenkopf (2012) also observe that despite the growth in the literature of research projects that compare the extent and forms of the political incorporation of immigrant communities, the field “still lacks comprehensive information on immigrant electoral outcomes and political antecedents across a large number of settings” (p. 21).

In addition, we discern a notable lack of data-driven reviews in this type of research, which can serve to complement and synthesize results from the growing pool of both qualitative and quantitative studies in localized contexts on the theme of immigrant political representation. More broadly, this study sheds light on cross-contextual factors that determine the election of immigrants. While such a meta-review approach is relatively uncharted territory for research on this theme, the synthesis of findings from an expansive array of studies allows for a detailed understanding of determinants of immigrant candidate election outcomes in a way that can improve strategies of how marginalized groups seek representation in various jurisdictions. The present manuscript is unique because it employs a secondary data-driven approach to comprehensively identify and evaluate the factors that influence the election of immigrants to political roles in local government. Drawing on the data and conclusions from forty four studies representing a range of geographical areas and using a wide variety of methods, more than fifty distinct elements are identified. We provide details on the most salient factors across all of the studies included in this review, together with a discussion of the policy implications for practitioners and potential areas of inquiry for researchers.

## Methods

### Search Strategy and Screening

The review is guided by the following research question: *What factors influence the election of immigrants to political roles in local government?* The study employs the scoping review methodological framework presented by Arksey and O’Malley (2005), which includes a six-stage process that, instead of being guided by a highly-focused “research question that lends itself to searching for particular study designs (as might be the case in a systematic review), … is guided by a requirement to identify all relevant literature regardless of study design” (p. 22). The unique benefits of scoping reviews are particularly reflected in the latter stages in which the data are charted, collated, and summarized, which result in both a descriptive and numerical synthesis of the data and an analysis of the themes that naturally emerge.

Owing to the particularities around the semantics of terms related to migration, some clarification of the nomenclature was required in order to create an effective search string. The intended meaning of “immigrants” for the purposes of this study is individuals who are not born in the country in which they are seeking political candidacy. The United Nations has made a clear distinction in its formal definitions of “refugees” and “migrants,” with the former being “persons fleeing armed conflict or persecution,” while the latter “choose to move not because of a direct threat of persecution or death, but mainly to improve their lives by finding work, or in some cases for education, family reunion, or other reasons” (UNHCR 2016). Both definitions fit within the profile described in the research question and were accordingly captured in the search criteria. Another necessary distinction concerns the use of “immigrants” versus “minorities.” Despite the significant overlap in the populations being referred to when immigrants and minorities are studied in the literature, there are many cases in which the latter have resided in their home countries for generations. Including studies of minorities that have lived in societies together with a dominant majority population would have diverged from our purpose of trying to understand immigrants’ political candidacy as it relates to the processes of relocation and integration. Finally, since this review focuses on local elections, the search aimed to draw out studies exclusively examining electoral processes of lower levels of government.

Taking into account the aforementioned factors, the search string that was used is as follows: (immigrant* OR refugee*) AND elect* AND represent* AND local government*. The search accommodated all peer-reviewed journal articles, book chapters, and books published between 1990 and 2019 (when the search was conducted) in order to capture those studies that best reflect the contemporary flow of migrants in the world today. No jurisdictional or language limitations were specified.

### Study Selection Process

The search for relevant sources to include in this study was undertaken in three phases. The initial search was conducted in a number of electronic databases, including Geobase, International Bibliography of Social Sciences, Scopus, Social Science Citation Index, Sociological Abstracts, Web of Science, and Worldwide Political Science Abstracts. After duplicates were removed from the results of these seven databases, the titles of 25,271 unique records were screened for peripheral to direct relevance to the elements of the research question. The eighty seven sources that remained were abstract screened with additional rigor, and fifty two of those sources were read in full. Of these, twenty three contributions were included in the final study from this phase.

The screening process took in account various factors, drawing upon the logic informing the preparation of the search string as described above. Studies focusing on a minority population were included if the group in question is also experiencing a noteworthy contemporary migration flow to the destination country and were excluded if this was not the case. Additionally, those sources that focused exclusively on federal- or state-level elections or local elections for positions beyond those on municipal councils (i.e., not mayor or councilor) were not considered. Studies also had to draw on primary data collection methods—with no preference given to qualitative or quantitative approaches—or offer a substantial analysis of relevant secondary data. In instances where multiple studies published by a given author focused on the same case study and emphasized the same factors, the more comprehensive of the two sources was selected for the review. For the analysis, the initial identification of the factors was conducted by the first author. Subsequently, both authors extensively discussed and categorized the factors into the hierarchal organization presented in the following sections.

In addition to the initial search in the seven primary databases, two further steps were taken in the search process. First, the search string used in the initial phase was also processed in Google Scholar. The first 1,000 sources in the search results were simultaneously title- and partially text-screened, as Google Scholar highlights portions of the document text that match the query string when displaying the search results. The presentation of the results in this manner in Google Scholar also meant that they could not be extracted in a format that was compatible with the results that were collected from the seven primary databases in the first stage of the analysis, which is the primary reason as to why Google Scholar was not included in the initial phase of the scoping review. For this step, duplicate sources and those not relevant to the research question were removed. From the remaining selections, eleven sources were identified for abstract screening, and all were subsequently read in their entirety. Of these, six articles were included in the final study.

**Figure 1.**
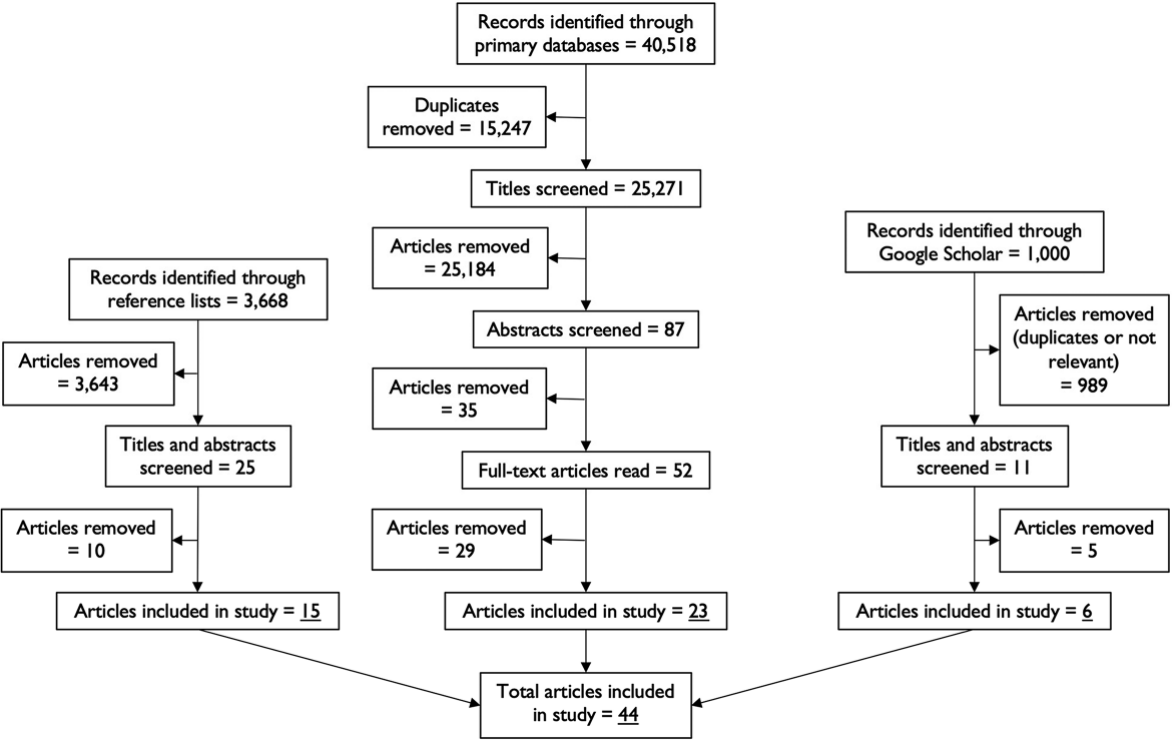
Summary of research methods employed for scoping review.

Second, the reference list of each source that was included in the review was also integrated into the search process. In this stage, the intention was to ensure that those sources that were repeatedly cited in relevant studies but did not emerge in the results of the searches in the seven primary databases or Google Scholar were also considered for inclusion in the review. The same process of title- and abstract-screening, as well as reading papers in full, was also undertaken to determine the relevance of studies for the review. In total, 3,668 titles were screened from reference lists (which included duplicates of those screened from the primary database and Google Scholar searches), from which twenty five were selected for abstract screening. All the sources were also fully read, of which fifteen were identified as relevant for inclusion in the study. A summary of the overall review process is presented in Figure 1.

## Results

### Summary of Selected Studies

**Figure 2.**
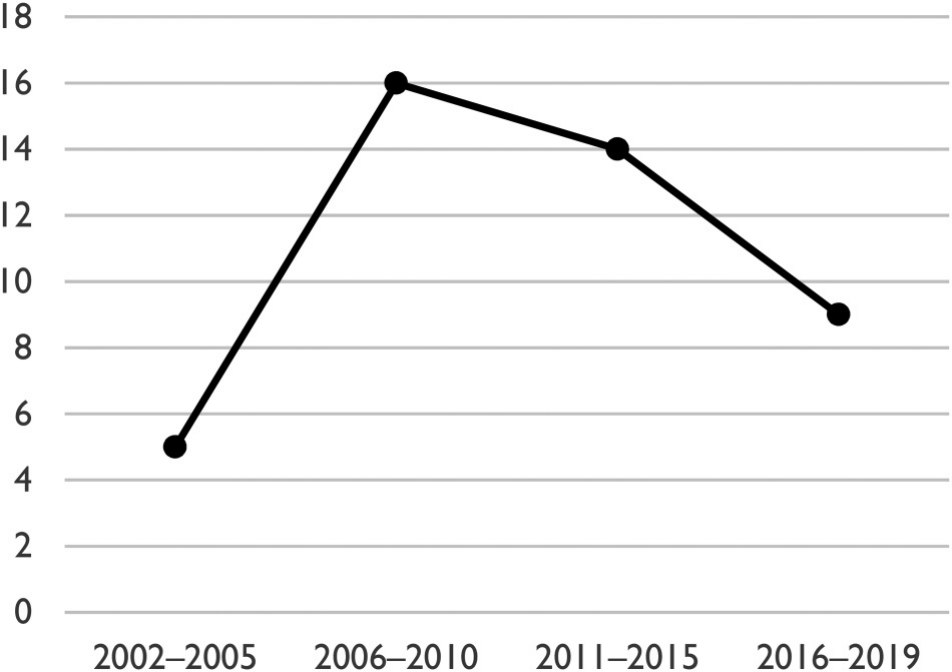
Distribution over time of publications included in study.

In total, forty four studies were included in the final analysis. There were twenty eight publications based on studies in Europe and twenty five focused on the North American context; a more detailed summary of the geographic locations covered by the sources that were selected can be found in Table 1. The studies employed both quantitative and qualitative methods to capture their findings and derive their conclusions. A quantitative approach—employing techniques such as bivariate and multivariate analyses—was used in thirty three of the studies, while qualitative data sources—featuring thematic analyses of in-depth interviews, focus group discussions, and case studies—were drawn upon for sixteen of them. A combination of both quantitative and qualitative methods was employed in five of the studies. One non-English source, in Spanish, was read (as the first author is fluent in the language) and included. Despite having specified a publication range starting at 1990, the studies included in the review ended up being published only between 2002 and 2019 (see Figure 2 for the distribution of publications per year over this period). While the number of unique studies released per year on this theme increased during the 2000s, it gradually subsided over the course of the 2010s. A comprehensive summary of the sources included in the scoping review is presented in Table 2.

**Table 1.** Geographic Locations of Studies Selected.

Continent	Country	No.
Europe	Other	6
	United Kingdom	5
	France	4
	Germany	4
	Denmark	3
	Ireland	3
	Netherlands	3
	Total	28
North America	Canada	16
	United States	9
	Total	25

**Table 2.** Summary of Articles Included in Review.

No.	Author(s) and year	Scope and key relevant findings for immigrant candidates’ electoral success
1	Barreto (2010)	Multiple data sources, including interviews with Latino mayoral candidates and support staff on election experiences in the United States
		Latino candidates tend to mobilize Latino electorate, regardless of issue positions and party preferences, especially through use of symbolic representation
2	Bergh and Bjørklund (2003)	Quantitative analysis of election of immigrant candidates in Copenhagen (Denmark) and Oslo (Norway)
		Immigrant candidates generally benefited from electoral systems using preferential voting ballots, which immigrant voters tend to use extensively to support such candidates
3	Bergh and Bjørklund (2011)	Bivariate and regression models on surveys of voter behavior in Norway
		Immigrant voters tend to indicate a strong preference for ballots featuring immigrant candidates, especially those of same nationality
4	Biles and Tolley (2008)	Survey of elected representatives on ethnic minority representation in Ottawa (Canada)
		Inhibiting factors for immigrant candidates include limited access to economic capital for financially supporting campaigns and comparative success of non-immigrant candidates owing to incumbency
5	Bird (2005)	Qualitative case studies of ethnic minority representation in Canada, Denmark, and France
		A broad array of factors that can both facilitate and hinder success for immigrant candidates, ranging from extent to which integration is prioritized in citizenship regimes, to parties’ inclination to put such candidates on their ballots, to impact of intra-/inter-ethnic affinity voting, among others
6	Bird (2008)	Questionnaire of elected representatives on ethnic minority representation in Hamilton (Canada)
		A number of social and human capital characteristics that limit opportunities for immigrant candidates, including economic and living conditions of marginalized communities and reduction of electoral seats available following municipal amalgamation
7	Bird (2015)	Focus group interviews with ethnic minority communities on views relating to minority representation in Toronto (Canada)
		Preferences regarding immigrant candidate political representation are contextually derived, with key factors including intra-ethnic affinity voting and candidates’ use of symbolic representation
8	Bird et al. (2016)	Multivariate analysis of survey of eligible voters examining interactive effects of gender and ethnicity in Toronto (Canada)
		Indications of intra-/inter-ethnic affinity voting in support of immigrant candidates, as well as diverging results relating to support for minority women candidates among certain voter groups
9	Bloemraad (2006)	Interviews with immigrant individuals associated with newcomer settlement in Toronto (Canada) and Boston (the United States)
		Wide-ranging factors impact immigrant candidates' success, including candidates' education levels, immigrant community resources, and strength of community organizations' infrastructures, among many others
10	Bloemraad (2008)	Proportionality analysis of election of ethnic minority candidates versus municipal population in Vancouver (Canada)
		Advantage provided to immigrant candidates through ethnic concentrations by capitalizing on ethnic affinity voting as well as presence of a municipal party system, while “at-large” (i.e., no districts/wards) electoral model can prevent success
11	Boudreau, Elmendorf and MacKenzie (2019)	Quantitative analysis of surveys of eligible voters and exit polls and effects of ethnicity and ideology on voters' choices in San Francisco (the United States)
		Ideology influences voters' choices in multi-ethnic elections, but ethnic group endorsements weaken spatial voting, as they can deter non-minority voters from supporting immigrant candidates
12	Casellas and McBrayer (2019)	Probability analysis of election of minority candidates in forty cities (the United States)
		Gentrification results in broad racial and demographic changes, such as an increase in ethnic minority populations in non-minority dominated districts, which works to detriment of immigrant candidates
13	Celis, Eelbode and Wauters (2013)	Probability analysis of selection of minority candidates for party ballots in Antwerp and Ghent (Belgium)
		Immigrant-community size is proportionally reflected in candidate lists, although not necessarily resulting in successful election outcomes for immigrant candidates; tendency of success of parties that candidates choose to represent appears to be most critical
14	Dancygier (2014)	Probability analysis of election of Muslim candidates in the United Kingdom
		Size of Muslim population and spatial concentration of community found to be most central to influencing share of Muslim immigrant candidates elected
15	Dancygier et al. (2015)	Regression analysis on election of immigrant candidates in Sweden
		While individual resources or structural variables were historically significant limiting factors, especially seats-to-voters ratios, discrimination by party gatekeepers by placing immigrant candidates in unfavorable party list positions appears to be most crucial
16	de Graauw and Vermeulen (2016)	Qualitative case studies of immigrant representation in Berlin (Germany), Amsterdam (Netherlands), New York City, and San Francisco (both the United States)
		Immigrant candidates' success impacted by community size and number of ethnic organizations, their effectiveness, and a relatively strong sense of a shared group identity between them, as well as candidates' prior work experience for them, among others
17	Fanning, Howard and O’Boyle (2010)	Interviews with immigrant candidates and party representatives on election experiences in Ireland
		Immigrant candidates' campaigns are most effective when championing local issues, while role of non-minority voter discrimination against such candidates can be downplayed
18	Garbaye (2005)	Interviews with politicians, party officials, and community organizers in Lille, Roubaix (both France), and Birmingham (the United Kingdom)
		Timing of relocation for immigrant candidates', their economic circumstances, citizenship regime of host state, and how political culture in their country of origin interacts with that of native political culture where they are seeking election are all importance factors for electoral success, among many others
19	Garcea (2008)	Questionnaire of elected representatives on ethnic minority representation in Regina and Saskatoon (Canada)
		Immigrant candidates benefit most from social capital—including extent to which they are connected to social, economic, and political networks in their communities—as well as access to run for seats that are considered to be “winnable”
20	Ginieniewicz (2010)	Individual and focus group interviews with Latino community on matters of representation in Toronto (Canada)
		Disunity within immigrant community, length of residence, their economic situation, and lack of effective leadership are considered as primary obstacles to increasing immigrant candidate representation
21	Hajnal (2009)	Simulations measuring impact of increased voter turnout on electoral outcomes in 20 cities (the United States)
		Beyond increasing number of seats per voting district, higher overall voter turnout, especially in larger cities with larger proportions of immigrant populations, is identified as most critical element for benefit of immigrant candidates
22	Maxwell (2013)	Quantitative analysis of proportional representation of immigrant communities in council memberships in 225 municipalities (France and the United Kingdom)
		Immigrant group mobilization—which can conversely be minimized by improved social integration, with demands becoming less urgent as their conditions stabilize—and spatial segregation/concentration are found to contribute to higher rates of immigrant candidate proportional representation
23	McGregor et al. (2017)	Logistic regression for success of hypothetical ethnic minority incumbent candidates based on survey of eligible voters in Toronto (Canada)
		Voter racial affinity appears to be critical for immigrant candidates' success, especially in wards where an incumbent candidate is not running
24	Michon (2011)	Interviews with ethnic minority politicians on political and election experiences in Paris (France) and Amsterdam (Netherlands)
		A broad range of factors working for and against immigrant candidates, including diverse electoral and party systems, presence and support of civil society organizations, and pre-electoral experience as a form of political legitimization to be drawn upon for launching candidacies, among others
25	Michon and Vermeulen (2013)	Quantitative analysis of ethnic organization density and council immigrant membership composition in Amsterdam (Netherlands)
		Immigrant candidates benefit from hailing from ethnic groups that have more tools to develop a robust civil society in host state will engage in active civic participation, gain greater access to political arena, and display overall higher levels of political engagement
26	O’Neill and Wesley (2008)	Questionnaire of elected representatives on ethnic minority representation in Winnipeg (Canada)
		Electoral systems, other political structures, and voter preferences, among other factors, present significant obstacles for immigrant candidates
27	Okigbo (2014)	Online survey of African voters and interviews/online surveys with African candidates on election experiences in Ireland
		Despite benefits of high pre-migratory educational attainment and command of local language, potential immigrant candidates' lack of political skills, resources, and information necessary for active political participation found in immigrant communities are barriers for candidacy launch or electoral success
28	Pérez-Nievas et al. (2014)	Thematic and statistical analysis of surveys of local organizations of political parties regarding immigrant candidates in Spain
		Immigrant candidates' community concentration in electoral district, candidates' cultural proximity to native population, and openness of political parties to include such candidates on their lists all increase likelihood for nomination, among other factors largely centered on party functioning
29	Portmann and Stojanović (2019)	Multilevel analysis of ballots cast relating to candidates elected in Zurich (Switzerland)
		Immigrant candidates with non-Swiss names receive more negative preference votes than those with typical Swiss surnames, including those whose names indicate origins in Western countries, with impacts becoming more pronounced when candidates run for Right or Center-Right parties
30	Reckhow (2009)	Regression analysis of minority politicians versus ethnic organization distribution in thirty cities (the United States)
		Immigrant candidate elected representation largely increases as a racial or ethnic group's proportion in population increases, although effect of advocacy organization density is not necessarily significant for Latinos
31	Sampert (2008)	Questionnaire of elected representatives on ethnic minority representation in Calgary and Edmonton (Canada)
		Immigrant candidates' success is hindered by a lack of residential concentration along ethno-racial lines, as this prevents mobilization of a sizeable voting minority community, as well as racist attitudes toward such candidates, together with other factors
32	Schönwälder and Kofri (2010)	Comparative analysis of minority politician distribution across twenty nine cities (Germany)
		A wide array of factors affecting immigrant candidates' fortunes, including solidified political party dynamics preventing access to new groups, low levels of immigrant concentration requiring candidates to draw support beyond immigrant vote, and a lack of immigrant community structures to foster increased participation, among others
33	Siemiatycki (2011)	Proportionality analysis of election of ethnic minority candidates in Toronto (Canada)
		Wide-ranging findings—including impacts of enlarged wards through municipal boundary revisions or “homeland imprint,” based on communities' minimal experience with liberal democracy prior to relocation, among many more—on factors dampening participation of immigrant groups in elections and in turn affecting potential rise and possible favorable outcomes for immigrant candidates
34	Siemiatycki and Saloojee (2002)	Survey of council members on identity politics in Toronto (Canada)
		Similar findings as above study (see Siemiatycki 2011), but found immigrant group home ownership rates as most important predictor of voter turnout, especially at municipal level where voting among tenants is historically low, in turn affecting immigrant candidate success
35	Simard (2008)	Questionnaire of elected representatives on ethnic minority representation in Montréal (Canada)
		Immigrant candidates' possible emergence and success impacted by ethnic group residential concentration, prior activity in community organizations, and extent of familiarity with local language
36	Sonenshein and Pinkus (2002)	Quantitative analysis of exit polls tracking voter profiles of ethnic minority candidates in Los Angeles (the United States)
		Immigrant candidates' appearance and triumph related to leadership required to coherently shape minority interests and formation of coalitions between and among minority groups, together with non-minority progressives, to advance those interests
37	Spicer, McGregor and Alcantara (2017)	Logistic regression analysis testing probability-of-success factors for candidates based on gender and ethnicity in twenty two cities (Canada)
		District magnitude is negatively associated with success of both female and/or immigrant candidates, pointing to multi-member districts harming their chances in absence of an established party system
38	Street and Schönwälder (2021)	Multivariate analysis testing probability-of-success factors for candidates based on ethnicity in four cities (Germany)
		Stigmatization of migrant-origin residents and tighter networks of ethnic organizations tend to result in demand for immigrant political representation and mobilize support for immigrant candidates
39	Szlovak (2017)	Interviews with politicians, candidates, party officials, and community organizers on election experiences in Ireland
		Immigrant candidates have an improved chance of being selected when their communities are inclined to show strong support for one party over others, although absence of blatant anti-immigrant rhetoric can dampen migrant communities' political mobilization in organizing protest votes
40	Tatari (2014)	Regression analysis and interviews with government officials and candidates on Muslim representation in London (the United Kingdom)
		High spatial concentration and deprivation—including in terms of income, employment, health and disability, education, skills and training, housing, living environment, and crime—of Muslim communities are correlated with more successful Muslim immigrant candidates
41	Thrasher et al. (2017)	Linear regression, χ^2^, and student's *t* analysis testing name discrimination from cast ballots and party candidate selection in the United Kingdom
		Outcomes of up to 5% of local elections are being decided by name discrimination against immigrant candidates, resulting in local parties calibrating their lists, by mitigating anticipated discrimination effect of name recognition, in internally selecting non-minority candidates
42	Togeby (2008)	Quantitative analysis of proportional distribution of minority politicians versus ethnic population density in Denmark
		A broad collection of findings impacting immigrant candidates, ranging from immigrant-supportive political opportunity structures, a combination of proportional representation and preferential voting resulting in parties vying to place immigrant candidates higher on ballots, and extent to which such candidates have established networks within their local communities, among others
43	Trounstine and Valdini (2008)	Regression analysis testing effects of electoral system on minority representation and interviews with politicians in the United States
		Once minority communities are highly concentrated and established as a large proportion of population, single-member district elections are found to generally aid immigrant candidates' electoral success
44	Vermeulen and Doğan (2015)	Interviews with Turkish-origin politicians on political and election experiences in Berlin (Germany)
		While immigrant candidates appear to have greater opportunities for selection when running for Left-wing parties, but having strong political connections, especially with influential non-immigrant members of their parties, is most decisive

### Factors Affecting the Election of Immigrant Candidates to Local Government

In total, fifty six unique factors were identified as consequential for the election of immigrants to local government positions (see Appendix 1). A critical theoretical element that underpins our hierarchical organization of the identified factors is the role of structure and agency as it relates to immigrant integration and empowerment. “Macro-,” “meso-,” and “micro-” level assessments are already prevalent in the discourse on the global movement of populations, especially in measuring patterns and processes of international migration (see Bloemraad 2013*b*; de Haas 2010; Massey et al. 1998). While the macro-level encapsulates those influential elements that are largely outside of the immediate control of an individual candidate, meso- and especially micro-level factors are much more within their realm of influence. Meso-level factors can be theorized as bridging structure and agency, as they are both subject to the force of existing structures while also having some ability to affect and reshape them, as well as contribute to the creation of new structures.

With this theoretical starting point, together with the insightful overview provided by Bloemraad and Schönwälder (2013), the hierarchy and subcategories into which fifty six factors were eventually grouped came into sharp relief. The three overarching categories include: 1. Macro-level electoral structures and situational elements; 2. Meso-level immigrant group dynamics; and 3. Micro-level individual candidate characteristics. This categorization provides the structure within which the most frequently identified factors are presented and discussed below. From the forty four sources included in our study, some addressed factors at multiple levels. We identified that thirty five of them addressed some macro-level factors, thirty six evaluated meso-level factors, and only twenty six studies explicitly addressed factors related to individual candidates’ characteristics at the micro level.

In this section, we present the nine most salient factors, which were determined based on the frequency of their appearance in the included studies. The adopted minimum standard for factors to be significant enough to warrant detailed attention and discussion was that they be identified in at least around 25% of all studies included in the review (i.e., in at least ten different sources). In addition to these, one or two additional noteworthy factors were identified for further analysis under each category. A summary of the salient factors discussed in this section, which also includes other notable factors that we have identified, can be found in Table 3.

**Table 3.** Summary of Salient Factors.

**Category**; Frequently-cited factor; *Noteworthy factor*
**Macro-level: Electoral structures and situational features**
Willingness of party leaders to include immigrant candidates in party lists or promote immigrant interests
Presence and/or size of municipal constituency boundaries
*Electoral ethnic discrimination*
*Institutional encouragement of newcomers and/or non-nationals to participate in local politics and/or elections*
**Meso-level: Immigrant group dynamics**
Concentration of immigrant populations in electoral districts
Ethnicity of immigrant candidates as it pertains to ethnic and/or inter-ethnic affinity
Degree of political unity among immigrant and/or minority community
Significance of economic adversities facing immigrant populations
Number, density, effectiveness, and sense of shared group identity of ethnic organizations
*Endorsements of ethnic organizations for immigrant candidates*
**Micro-level: Individual candidate characteristics**
Prior political party and/or civic activism of immigrant candidates
Immigrant candidates' economic wealth and/or access to resources
*Immigrant candidates' use of symbolic representation to draw support from immigrant groups*

### Macro-Level: Electoral Structures and Situational Elements

The factors categorized in this group fall under the following six subthemes: 1. The functioning and influence of political parties; 2. Electoral systems and structures; 3. Institutional architecture for immigrant political empowerment; 4. Geographic circumstances within municipalities; 5. The operations and characteristics of governments at all levels, including their treatment of immigrants; and 6. Other situational elements, such as ethnic discrimination and electoral turnout.

Given the significant role that parties play in politics today, it comes as no surprise that factors related to their function and operations are mentioned in two-thirds of the selected studies. Specifically, the willingness of party leaders to include immigrants in voting lists or to promote the mobilization of immigrant interest groups emerged as important determinants of the election of immigrants in these sources. The case studies presented instances in which party gatekeepers had both helped and hindered immigrants’ access to positions of candidacy. In Ghent and Antwerp (Belgium), for example, strong migrant political representation was linked to specialized efforts made by local parties to have immigrant candidates on their party lists, which naturally put them in a more favorable position for election (Celis, Eelbode and Wauters 2013). Conversely, exclusion from the electoral systems guarantees that immigrants have no chance of being elected to office. For example, some local parties in the United Kingdom anticipated that voters would discriminate against certain candidates based on name recognition and used this as justification to exclude immigrant candidates as electoral candidates in their internal selection processes (Thrasher et al. 2017).

Another feature of elections that was found to be of considerable influence is the presence and size of municipal constituency boundaries. In the case of Toronto (Canada), the districts used in the ward system were viewed to inherently minimize the possibility of an immigrant candidate to emerge or for an immigrant voting group to have a substantial impact because the wards were too large and too diverse (Siemiatycki 2011). One study of Latino, Asian-American, and other immigrant and/or ethnic minority candidates in the United States also concluded that limiting the number of seats on council increased the threshold for the number of voters required to control a seat, dampening the effect that minority voters could have on the number of seats held by immigrants (Hajnal 2009).

One noteworthy factor that did not feature heavily in the selected studies was ethnic electoral discrimination against immigrant candidates, largely because it is a difficult phenomenon to quantify. Nevertheless, one study was able to capture this owing to a particularity of the Swiss cantonal election process, which allows for voters to not only rank their preferred candidates but also cast “negative” preference votes against those candidates that they do not wish to support (Portmann and Stojanović 2019). An analysis of actual ballots cast by voters in Zurich determined that immigrant-origin candidates with non-Swiss names, including those with Western names from other nationalities, suffered an electoral penalty.

Another notable factor that appeared to loom over the ability of immigrant candidates to develop the capacities required to be able to endure the grueling conditions of local elections was the extent and manner in which institutions encouraged the participation of newcomers and non-citizens in local politics. Just as local governments can discourage immigrant political incorporation by employing exclusionary policies, such as those disenfranchising their non-citizen residents from voting in municipal elections, they can also play a crucial role in promoting newcomer political inclusion both within and outside of the context of electoral processes. One study of Roubaix (France) described the commitment of the municipal government to the notion of the city's inhabitants (over its “citizens” or “voters”) as political actors by institutionalizing community movements within its “*comités de quartier*” (i.e., “neighborhood committees”). The empowering environment found in these spaces would lead to establishment of a North African political core that began to gain prominence in local elections in the 1990s (Garbaye 2005).

As was described earlier, the macro-level factors presented here are essentially outside of the control of the candidates and depend on the context in which immigrants choose to run for political office. Yet, the two outstanding factors discussed here—namely, the role played by political parties in determining whether or not immigrants can run as candidates and the impact of the presence and size of electoral boundaries—could still be addressed through broader, long-term strategies, such as diversity quotas or structural reforms to account for those elements that harm the electoral chances of immigrant candidates.

### Meso-Level: Immigrant-Minority Group Dynamics

The factors found in this group relate to: 1. General immigrant-minority group dynamics; 2. The nature of electoral participation of immigrant groups; 3. Electoral mobilization of immigrant groups, particularly as it relates to being marginalized; and 4. The influence of ethnic organizations.

Building on the previously-mentioned factor of the size of municipal constituency boundaries, a higher concentration of immigrant populations in electoral jurisdictions was seen as a considerable asset for immigrant candidates. The interplay between the two factors is best exemplified in a study from the United States, where the district system was understood to increase diversity on city councils only when underrepresented groups were highly concentrated and made up a substantial portion of the population (Trounstine and Valdini 2008). Similarly, in the United Kingdom, South Asians’ spatial segregation and concentration in electoral districts gave them leverage as British political parties increasingly looked to candidates from their communities as a reliable way to win seats on municipal councils (Maxwell 2013).

As anticipated, the ethnicity of immigrant candidates as it pertains to ethnic and inter-ethnic affinity has proven to be very influential in turning out migrant and/or minority voters in those candidates’ favor. In the Chow candidacy featuring in the Toronto mayoral election that was discussed earlier, there was strong evidence of an inter-ethnic affinity effect among minorities for an immigrant candidate, although it did not sufficiently insulate her campaign from its eventual loss (Bird et al. 2016). In Norway, irrespective of background, most voters were found to support ballots with immigrant candidates, but immigrant voters were observed to do so to a markedly greater extent (Bergh and Bjørklund 2011).

Immigrant candidates’ attractiveness to a particular voter group, and especially their own, was found to largely depend on the degree of political unity within that immigrant and/or minority community. One prominent study attributed the relatively strong presence of members of the Pakistani community on local councils in the United Kingdom, in comparison to other migrant populations, to a combination of factors, but highlighted their identity as representatives of their ethnic constituencies’ collective interests as a key element (Garbaye 2005).

Economic adversities faced by immigrant populations was characterized as a barrier to immigrant candidates’ political success in two ways: this was either in terms of how likely they were to overcome financial challenges associated with the election processes, or how the economic fortunes of groups to which they belong are projected on immigrant candidates in the context of elections. Another example from the United Kingdom, this time focused on three boroughs in London, points to a feedback loop of the weak substantive representation of Muslim elected officials, who are demonstrated to be the primary advocates of Muslim interests through that study's analysis, perpetuating the further disenfranchisement of an already socio-economically disadvantaged minority and prolonging the exclusion of Muslims from elected and appointed office (Tatari 2014).

Finally, ethnic organizations featured prominently in the selected sources for this review. Studies considered a number of elements related to ethnic organizations, including their number and density within a given jurisdiction (e.g., Reckhow 2009) and their coherence and a sense of shared group identity (or lack thereof) among them (e.g., de Graauw and Vermeulen 2016). In Amsterdam (the Netherlands), the dense organizational infrastructure of the Turkish population was linked to its success in gaining positions of elected office versus the Moroccan population, which was described as having a more “individualist” integration model (Michon and Vermeulen 2013). Despite the generally positive light in which ethnic organizations were cast, their endorsements for immigrant candidates were also found to have unintended negative consequences. Research in the United States discovered that while ethnic group endorsements were likely to result in members of those groups supporting those candidates that the organizations had supported, it also served as a deterrent for White voters (Boudreau, Elmendorf and MacKenzie 2019).

In summary, these meso-level characteristics highlight areas over which immigrant communities and individual candidates have some degree of control. In particular, the degree of political unity within migrant communities and the role of ethnic and/or inter-ethnic affinity in voting for immigrant candidates are realities that immigrant communities, based on their level of organization, can strive to foster. As seen above, a host of characteristics relating to the presence and influence of ethnic organizations can serve to significantly advance immigrant candidates’ electoral prospects, although there are exceptions.

### Micro-Level: Individual Candidate Characteristics

The factors at the individual- or micro-level generally relate to the following subthemes: 1. Immigrant candidates’ prior political and/or civic experience; 2. Characteristics related to how they have integrated into society; 3. Candidates’ approaches to campaigning, especially as it concerns their relationships with political parties and the migrant population; and 4. Immigrant candidates’ general attributes, namely education and gender.

At least 25% of the studies that were included pointed to immigrant candidates’ economic wealth and/or access to resources as critical for their electoral success—reinforcing similarities with the “meso-level” factor described above regarding economic adversities confronting migrant populations. In Spain, despite a relative advantage enjoyed by Latin American immigrants because of the affinity of the cultures in their countries of origin with the local culture, European migrants were still more successful in gaining political candidacy owing to their comparatively higher average socio-economic status (Pérez-Nievas et al. 2014). This also applied in the context of exorbitant campaign costs and was presented as a possible barrier that disproportionately affected immigrants in Ottawa (Canada) wishing to enter the electoral arena (Biles and Tolley 2008).

Prior activism of immigrant candidates—either generally in terms of civic leadership, especially in local immigrants’ rights organizations, or in political parties—emerged as highly consequential. A survey of immigrant candidates in Ireland found that acquiring political capital, largely through intensive political activity and developing strong networks in the local area, was a central factor for successful selection (Szlovak 2017; see also Okigbo 2014). One study of immigrant candidates in Amsterdam described how they felt legitimatized to stand in an election owing to their political “socialization.” This was often gained through acquaintanceship with other representatives through family, friends, or civic activities, and in turn increased access to knowledge beneficial for navigating the political arena (Michon 2011).

Another factor associated with immigrant candidates’ campaigning behavior was the extent to which they drew support from migrant groups through the use of “symbolic representation,” which is another form of representation referred to by Pitkin (1967). Employing this form of representation entails using language or participating in religious or cultural ceremonies to signal solidarity with or indicate recognition and respect for a particular community (Bird 2015). A study in the United States points to how Latino candidates employ the notion of “*nuestra comunidad*” (i.e., “our community”) to not only connect their agenda to the larger goals of the Latino community but to also strengthen the sense of comradeship between the candidates and the community (Barreto 2010).

The factors found under this category point to those elements over which immigrant candidates largely have control and concerning which they are able to exercise agency, especially as it relates to how they can position their campaigns. From within this group of factors, prior experience in terms of activism in political parties and/or civic affairs and use of “symbolic representation” seem to be most critical in positive election outcomes. Of course, other factors associated with immigrant candidates’ characteristics, such as their economic wealth and/or access to material resources, are not in the full control of immigrant politicians. Nevertheless, factors addressing access to resources point to systemic imbalances within electoral structures, most notably concerning prohibitively expensive campaign-related costs.

## Discussion and Conclusion

### Discussion

The defining feature of the classification system employed in presenting the findings is anchored in the interplay between electoral structures and individual and collective agency. In the context of immigrant candidates’ electoral success, macro-level factors influence the process of incorporation, serving to facilitate or block their actions, while micro-level factors are largely in potential candidates’ control and allow them to chart a course of their choosing. In between these two layers is the meso level, with the factors at this scale functioning as a bridge through which individuals and communities can contribute to shaping and are also simultaneously shaped by the macro-level factors. Highlighting the potential of the meso and micro levels, Hochschild et al. (2013) explain that “[i]mmigrants are most likely to attain initial political success by mobilizing as groups, but full incorporation is achieved only when individuals feel free to choose whether to meld into mainstream society or remain closely linked to their group” (p. 21). Each route naturally has its own benefits, with candidates likely wishing to mainstream when running in electoral districts that are either majority-population dominated or feature a breadth of diverse communities, and perhaps deciding to emphasize their ethno-racial ties when contesting seats located in voting precincts that overlap with enclaves centered on the migrant group to which they belong. In this connection, Berry’s (1997) discerning typology on immigrant acculturation considers “integration,” which combines mainstreaming with the preservation of ethnic ties, as the most successful path, demonstrating that choosing to mainstream or maintaining one's ethnic ties are not mutually exclusive options.

Our assessment indicates that researchers have been studying an extensive range of factors at the macro level in comparison to the other two levels. Most studies of macro-level factors focus on electoral systems and their relation to immigrant political success (e.g., proportional representation, preferential voting, majority-vote, the presence of incumbency, etc.). Also, a large number of studies at the macro level look at the role and operations of political parties, which, given their presence in most municipal elections, nevertheless appear to be highly consequential. This suggests that researchers perceive macro-level factors as the most important determinants of immigrant electoral fortunes. Parties tend to suffer from rigid and hierarchical structures, resulting in dense barriers for potential immigrant candidates to break through (Celis, Eelbode and Wauters 2013). Further, if candidates are not selected to a party list and choose to run as independents, they are deprived of critical benefits (largely financial in nature) that can starve and essentially condemn their campaigns. Parties that find that their investments in the migrant population are not generating results may pull away from them as viable candidates or as reliable voters for subsequent elections (Szlovak 2017), with those groups suffering longer-term impacts owing to the length of electoral cycles. This principle can be expanded beyond political parties to the broader institutional architecture that facilitates immigrant political empowerment, which can apply to the federal down to the municipal levels of government. Public funding efforts dedicated toward immigrant integration, which can range from supporting non-governmental community-building efforts to providing in-state tuition, should be treated as investments in their own long-term socio-economic well-being (Jones-Correa 2011), as well as a means of protecting the enduring integrity of their democratic systems. It is important to recognize that while changes at the macro level are not necessarily impossible, they may be among the most difficult to effect due to the deep-rootedness of practices associated with such systems.

While candidates and communities are perhaps best served to focus on those factors over which they have greater control, there are nevertheless certain macro-level factors that appear to be most critical in hindering immigrant candidates’ success which should be targeted for change in the long term. With respect to electoral systems, these include increasing the number of electoral seats within a municipality, using proportional or preferential representation, and imposing regulations to even the playing field for campaign-related expenses. As it relates to political party functioning, some examples are challenging inflexible hierarchies, promoting viable immigrant candidates (in some cases, despite anticipated voter discrimination), and advocating for issues of significance for migrant communities.

The meso-level factors identified in this study can be theorized as bridging the structure of the political system and the agency of immigrant candidates. In particular, meso-level factors can be opportunities and conduits through which migrant communities facilitate and empower their members to arise as potential candidates. Community dynamics are connected in various ways to eight of the nine salient factors presented in this paper (as well as in at least two-thirds of the full list of factors, as presented in Appendix 1). The selected studies indicate that for a sustainable trend to be maintained in the direction of increased descriptive representation, a strong community presence is essential, as this has an impact on the two critical elements in elections: candidates and voters. Broadly speaking, emphasis on empowerment leads to increased social integration, which can then result in a better informed and, consequently, more mobilized population. In the context of elections, this can produce not only a larger number of better-prepared candidates, but also an enthusiastic community on which those candidates can depend at the ballot box—a decisive factor in elections if that population is sufficiently concentrated within a given electoral boundary. Communities’ strengths also appear to be closely related to the vigor of the support they receive from migrant and/or minority organizations. Such organizations provide environments that offer practical opportunities for immigrants to build capacity for effective political and social action, which has proven to be a key element in candidate preparedness and public appeal (Michon and Vermeulen 2013).

At the micro level, there are certain factors (e.g., level of education, economic wealth, etc.) that are somewhat out of the control of immigrant candidates at the time when they decide to launch their campaigns, although these have been proven to have similar impacts on non-immigrant candidates as well, albeit to varying degrees (see e.g., Siemiatycki and Saloojee 2002; Michon 2011; Dancygier et al. 2015). Beyond this, however, immigrants have free reign to determine where they wish to declare candidacy and what kind of campaign they wish to run, which appear to be most important for their electoral success. They can choose to champion the issues of their own communities, or even expand that to all migrant and/or minority populations, and they can also place emphasis on local issues in order to appeal to a broader base. Immigrant candidates are able to take advantage of approaches such as the use of symbolic representation to signal recognition and respect of their communities’ interests and also choose to engage in meaningful dialogue amongst diverse groups, perhaps reaching populations to which other non-immigrant and/or non-minority candidates may not have access (Bird 2015). Their prior experience in civic or political affairs is a factor that appears to be valuable in attracting voters from within and beyond their communities, as does the extent to which they have integrated into the societies of the countries in which they are seeking election. The critical point here is that, despite so many structural and systemic factors working against them, there is still a considerable degree of autonomy that immigrant candidates can exert in charting a path to electoral success. Thus, the concurrent interplay between actions and initiatives at the individual level and factors at the meso and macro levels reemphasizes the complexities associated with the fortunes of immigrants in local elections—thereby requiring dynamic policy actions directed at the individual level through to broader societal levels.

### Limitations

As with any review of this nature, recognition of its limitations is important and may assist with future related research. One element pertaining to the literature is the lack of uniformity in the nomenclature used to describe the populations being studied. Rather than solely relying on the terms “immigrant” or “minority” as they were employed in the sources, the review required a rigorous analysis to determine exactly the nature of the groups being evaluated. As a result, studies that may have been potentially relevant could have been overlooked in the title- and abstract-screening stages of the review. Incongruency also applies to the methods of study that were used. Both quantitative and qualitative approaches were taken into account, and even within these two categorizations, there was a substantial degree of variation in how the data was collected and assessed, reducing the effectiveness and reliability of the results to conduct comparisons. In addition, there is always a degree of subjectivity and bias in how findings are determined and presented, and this is particularly the case with qualitative studies and the way in which researchers conclude that a given factor was consequential.

In this connection, another issue associated with selection is whether the frequency with which a factor is mentioned in the literature serves as an adequate indicator of its importance. This approach served as the basis for identifying the salient factors to receive further attention in the presented results. It is important to note that the number of times a factor featured in the selected literature does not necessarily demonstrate that factor's robustness or greater strength in its explanatory power. This is especially the case if the studies that informed the analysis have not set out, in their own right, to comprehensively delineate the most significant determinants of immigrants’ electoral success for local government positions. Rather, the use of this somewhat elementary metric allowed for a straightforward identification of the factors to inform the qualitative overview offered by this paper, which serves essentially as an aid for the reader's benefit to assist with gaining a more nuanced understanding of the complete list of factors found in Appendix 1.

In our methodology, if a particular factor was evaluated and shown to be immaterial in a given study, it was rejected for inclusion in this review; future reviews of this nature may benefit from including studies on all potential factors, whether they are proven to be significant or not, as they may appeal to and could inspire researchers to test these conclusions in other contexts. Further, despite not having imposed a jurisdictional limit, all of the studies that were identified as relevant to the research question and subsequently selected for the study were carried out in Europe and North America. A more focused analysis into whether inquiries of this nature have been pursued in other jurisdictions could assist with gaining an increasingly global perspective on the electoral fortunes of immigrants in destination countries.

### Implications and Conclusion

The findings of this review shed light on potential areas of research that merit further attention with respect to the conditions leading to the election of immigrants to positions in local government. In particular, more research is needed to determine their implications in a wider variety of settings, especially in the Global South and outside of contexts that largely fall within the Western Liberal Democratic paradigm, which could point to potential factors relating to the influence of regimes and political systems in this regard. Additional themes that require examination include how the structures of electoral systems impact immigrant candidates’ likelihood of electoral success, the techniques that political parties can use to foster immigrant candidate emergence, and other potential means and approaches that candidates have at their disposal to nurture ethnic and inter-ethnic affinity and inspire migrant voter turnout. There is also a need for tracking how immigrant representation has changed over time, as regimes managing migration flows and practices around integration continue to evolve (Bloemraad 2013*a*). As indicated earlier, research on micro-level factors lags behind investigations into factors at the other two levels and should also be explored further. Given the constantly changing dynamics in migration flows to destination countries, more studies focusing on micro-level factors will illuminate our understanding of the broader evolution in migration and integration policies that are becoming manifest in political representation.

As it concerns policy and practice, our study points to a strong need for dedicated institutional involvement in immigrants’ political engagement and empowerment, including a greater emphasis on government- (and perhaps party-) supported civic education to increase general political awareness and engagement. Offering immigrants further opportunities to build capacity for participation in political and social action, especially in terms of inclusion in design and delivery of programs to meet their own collective needs, would result in better-engaged communities and community members. Given how well-positioned they are to carry out this work effectively, migrant- and/or minority-supporting organizations can play a critical role in contributing to the emergence of viable candidates and mobilizing significant blocs of voters, and fostering closer collaboration among these organizations would only reinforce their effectiveness. More broadly, electoral systems require ongoing scrutiny to determine how they can reflect greater openness and fairness, including through drawing constituency boundaries that are increasingly representative of the wards or districts that politicians are meant to serve and allowing non-naturalized immigrants to vote in local elections. Finally, governments may wish to evaluate their overall outlook toward immigrants and identify how they can best foster an institutional environment in which citizenship and ethnicity are prioritized in order to integrate newcomer populations more comprehensively.

This scoping review presents the findings of forty four studies investigating the factors influencing immigrant representation in local government. This data-driven study identifies a wide array of fifty six unique determinants and presents them in a hierarchical structure that emphasizes the role of individual and collective agency in how immigrants are elected. The research suggests that while broader structural factors continue to have considerable sway on immigrant representation, potential candidates, supported by the communities they hail from, also have a significant role to play in determining their own electoral success. At a time when questions abound over how democratic values can aptly respond to rapidly-evolving demographics in major immigrant-receiving countries, this inquiry into one aspect of its descriptive component underscores the significance of the broader question of representation.

## Appendix

**Table:** 1. Summary of All Factors by Category.

Category; subcategory	Factor
Macro-level: Electoral structures and situational features	
Political parties	Willingness of party leaders to include immigrant candidates in party lists or promote immigrant interests
	Internal party structures and established claims to positions of influence
	Presence and strength of political parties in electoral system
	Degree of polarization among political parties
	Presence of ethnic minority networks and/or subsections of ad hoc working groups within political parties
	Existence of regional and/or non-statewide political parties
	Whether candidates are seeking positions both through a constituency and on a party list (for mixed proportional representation and majoritarian electoral systems)
Electoral systems	Use of proportional representation in electoral system
	Presence of a majority-vote electoral system using closed lists
	Presence of incumbency in electoral system
	Use of preferential voting in electoral system
	Use of multi-member voting in electoral system
Institutional immigrant empowerment	Institutional encouragement of newcomers and/or non-nationals to participate in local politics and/or elections
	Eligibility of non-citizens to vote and/or run as candidates
Geographic conditions	Presence and/or size of municipal constituency boundaries
	Density of residential area within municipality
Government operations/ characteristics	Trends in appointment of immigrants to other government positions
	Share of electoral seats already held by immigrants
	Presence of power-concentration measures in local politics
	Degree of government progressiveness with respect to identity politics
	Nature of institutional environment welcoming newcomers
Other situational elements	Electoral ethnic discrimination
	Changes in tolerance among native and/or majority population
	Rate of overall voter turnout
Meso-level: Immigrant group dynamics	
Group dynamics	Concentration of immigrant groups in electoral districts
	Rate of naturalization among immigrant groups, including nature of assimilationism
	Timing of migration flows of immigrant groups
	Rates of home ownership among immigrant groups
	Other aspects of immigrant group social integration (e.g., cultural and religious practices, intermarriage rates, etc.)
General electoral turnout	Ethnicity of immigrant candidates as it pertains to ethnic and/or inter-ethnic affinity voting
	Degree of political unity among immigrant/minority groups
	Rate of voter turnout among immigrant groups
	Familiarity and/or comfort of immigrant groups with elections
	Transnational preoccupation of immigrant groups with politics in countries of origin
Marginalization-related electoral mobilization	Significance of economic adversities facing immigrant groups
	Concentration of immigrant groups in social housing “ghettos"
	Perception of being stigmatized and/or marginalized of immigrant groups
	Political Displacement Hypothesis (i.e., voter turnout of immigrant groups affected by demographic changes generating a perceived threat)
Ethnic organizations	Number, density, effectiveness, and sense of shared group identity of ethnic organizations
	Endorsements of ethnic organizations for immigrant candidates
Micro-level: Individual candidate characteristics	
Prior political/civic experience	Prior political party/civic leadership/activism of immigrant candidates
	Prior involvement of immigrant candidates in local immigrant rights organizations
Integration-related characteristics	Economic wealth and/or access to material resources of immigrant candidates
	Mastery of native language of immigrant candidates
	Length of residence of immigrant candidates
	Employment status of immigrant candidates
	Degree to which immigrant candidates are assimilated
	Proximity of immigrant candidates' culture to native culture
	Regional proximity of immigrant candidates' country of origin to country in which they are running for election
Campaigning behavior	Degree to which immigrant candidates are inclined to champion issues of their own communities
	Immigrant candidates' willingness to run in elections as representatives of larger political parties
	Immigrant candidates' tendency to represent leftist parties
	Immigrant candidates' ability to elicit support through emphasis on local issues
	Immigrant candidates' use of symbolic representation to draw support from immigrant groups
General attributes	Level of education of immigrant candidates
	Gender of immigrant candidates
